# Early autism risk factors: a cohort study of children born very preterm at 5-years

**DOI:** 10.1038/s41372-026-02856-x

**Published:** 2026-08-12

**Authors:** Meg Stone-Heaberlin, Allison D. Blackburn, Leanne Tamm, Armin Allahverdy, Beth Kline-Fath, Nehal A. Parikh

**Affiliations:** 1 Department of Pediatrics, Cincinnati Children’s Hospital Medical Center, Cincinnati, OH, USA.; 2 Department of Pediatrics, University of Cincinnati College of Medicine, Cincinnati, OH, USA.; 3 Neurodevelopmental Disorders Prevention Center/Perinatal Institute, Cincinnati Children’s Hospital Medical Center, Cincinnati, OH, USA.; 4 These authors contributed equally: Meg Stone-Heaberlin, Allison D. Blackburn.

## Abstract

**OBJECTIVE:**

Explore the association of prenatal, perinatal, and postnatal risk factors with autism risk at 5-years corrected age in very preterm (VPT) children.

**STUDY DESIGN:**

Data from a longitudinal cohort study of 315 VPT and 172 term-born children at a US-based academic medical center were analyzed, exploring antecedents of autism risk using elastic net regression.

**RESULTS:**

More children born VPT (8.9%) had a Social Communication Questionnaire score associated with autism (i.e., SCQ ≥ 15) than term-born controls (2.9%; *p* = 0.012). For VPT children, cerebellar abnormalities at term-equivalent age (OR = 1.339, 95% CI = 1.010–1.778) and moderate-severe histologic chorioamnionitis (OR = 4.148, CI = 1.294–13.368) were significantly associated with autism risk, in addition to male sex and social risk. VPT children with SCQ ≥ 15 had poorer adaptive, behavioral, cognitive, and executive functioning (all *p*s < 0.001).

**CONCLUSION:**

Early screening for children with these risk factors can facilitate early intervention for those at most risk for poorer neurodevelopmental outcomes.

## INTRODUCTION

Autism is a neurodevelopmental disability characterized by impairments in communication, reciprocal social interaction, and restricted/repetitive behaviors or interests. Children born prematurely are at risk for neurodevelopmental disabilities, behavioral and social difficulties, and impaired executive functioning (EF) [1], all characteristic of autism [2]. Perhaps unsurprisingly, autism prevalence in premature children is high, ranging from 7% to 28%, depending on type of assessment, degree of prematurity, and child’s age [3].

Children born very preterm (VPT) are susceptible to various prenatal, perinatal, and neonatal factors that may adversely affect brain development and contribute to autism risk [4]; however, there is no consensus regarding which early risk factors are prognostic, and there is variability across studies regarding which risk factors are included and how long children are followed. Additionally, large cohort studies in the United States (US) are rare, precluding generalizability of international study findings. As autism is highly prevalent in the US with significant personal, familial, and economic impacts [5], understanding autism risk in VPT children is crucial.

Here, we leverage data from a large, prospective US study to explore the association of various infant and maternal risk factors with autism risk in children born VPT (≤32 weeks’ gestational age). Although we did not have data regarding whether children met criteria for a clinical diagnosis of autism, data from the Social Communication Questionnaire (SCQ) [6] were available. The SCQ, empirically derived from the Autism Diagnostic Interview–Revised, has been demonstrated to be an effective screening instrument for autism (area under the curve = 0.885) after the age of 4 [7]. Higher SCQ scores are associated with elevated autism traits and symptoms and a score ≥15 is used to differentiate children with autism from other diagnoses (sensitivity 85%, specificity 75%), including non-autistic intellectual disability (sensitivity 96%, specificity 67%) [6]. The established cutoff of ≥15 has been further validated for discriminating children with and without autism in the general population and in VPT samples [8–10] (sensitivity 82%, specificity 88% [9]). Multiple prenatal characteristics were collected prospectively as well as delivery and neonatal characteristics before hospital discharge. The sample is being followed longitudinally, with the 5-year corrected age visit recently completed. Possible risk factors for autism that are available in the dataset include diabetes, hypertensive disorders of pregnancy (HDP), chorioamnionitis, Apgar scores, retinopathy of prematurity (ROP), bronchopulmonary dysplasia (BPD), sepsis, brain abnormalities, and breastfeeding status. We briefly review these risk factors next.

Pre-gestational/gestational diabetes has been associated with a 25% higher autism risk in term-born children [11]. Preterm birth may partially mediate the diabetes-autism link, accounting for ~20–40% of the association [12]. HDP are associated with a small yet significant increase in the risk of autism (including autism-related symptomatology and diagnoses) in term-born children [13]. Chorioamnionitis, i.e., inflammatory lesions in the chorioamniotic membranes of the placenta, has been identified as a risk factor for poor cognitive and motor outcomes in infants, placing them at higher associated autism risk [14]. Low Apgar scores have been consistently associated with higher risk for a variety of neurological and psychiatric disorders, with a low 5-minute Apgar score associated with increased autism risk [15]. ROP has been associated with cognitive impairment, cerebral palsy, behavioral problems, and neurodevelopmental impairment, including autism evaluated by “any validated tool” [16]. Extremely preterm children with moderate-to-severe ROP had a threefold increased risk of an autism diagnosis compared to those with no or mild ROP [17]. BPD is associated with later neurodevelopmental impairment, with one study indicating that 20% of VPT infants with BPD later received an autism diagnosis by parent report [18]. BPD may serve as a proxy for overall neonatal vulnerability rather than a direct causal factor for autism, but it underscores the need for follow-up in this high-risk population. A consistent association between neonatal blood stream infection and autism risk is reported in the literature [19]. Breastfeeding may be a protective factor for autism. A meta-analysis found that children diagnosed with autism were significantly less likely to have been breastfed than children without autism [20], including in extremely preterm children with high autism risk [2]. Increased autism risk in VPT children may be due to brain injury or aberrant cerebral development. Infants born prematurely have smaller total brain tissue volume, including smaller white and gray matter volumes, and cerebellums, than term-born peers [21]. Brain abnormalities are implicated in autism risk in children born full term [22]; however, fewer studies have explored their association with autism in VPT children [23]. Extant literature suggests white matter and cerebellar abnormalities contribute to autism diagnoses [23, 24] in premature children.

### Current study

Collectively, these findings underscore the importance of considering multiple risk factors in understanding risk for autism (i.e., SCQ ≥ 15) in children born VPT. The objective was to explore the association of prenatal, perinatal, and neonatal factors with autism risk at age 5.

## METHODS

### Participants

VPT infants (*N* = 395) without known chromosomal or congenital anomalies affecting the central nervous system or with cyanotic heart disease were recruited from five level III/IV Midwestern NICUs between September 2016 and November 2019. This sub-study included 315 VPT children who participated in a 5-year corrected age assessment. Additional participants included 172 term-born children recruited at age 5 between October 2022 and July 2025. Table 1 reports participant characteristics.

### Measures

#### Autism risk.

As described earlier, caregivers completed the Lifetime SCQ [6], an empirically derived 40-item checklist assessing history and current characteristics of autism. The SCQ score was dichotomized such that a score of 0–14 was coded 0 and a score of ≥15 was coded as 1 (i.e., at risk for autism).

#### Prenatal, perinatal, and neonatal risk factors.

Clinical characteristics, pregnancy/delivery data, and NICU parameters were systematically recorded. These included gestational age (GA), post-menstrual age (PMA), birthweight, 5-min Apgar <5, chorioamnionitis, HDP, BPD, ROP, sepsis (culture-positive blood or cerebrospinal fluid infection), pregestational/gestational diabetes, and whether a child was receiving their own mother’s breastmilk at NICU discharge. Histologic chorioamnionitis was graded using Redline criteria [25] and considered moderate at stage 2 and severe at stage 3 [26]; most infants had funisitis (*n* = 276; 87.6%). HDP included hypertension or maternal systolic/diastolic blood pressure >140/90 on ≥2 occasions. BPD grade was defined based on degree of respiratory support at 36 weeks PMA [27]. ROP was diagnosed by ophthalmologists using the International Classification of ROP [28]. A social risk score, comprised of household income, maternal employment status and education, family structure, languages spoken at home, and maternal age at birth, was established for the VPT group at enrollment and for term-born controls at age 5 [29]. Each family was scored on the number and severity of social risk characteristics (range 0–12).

#### MRI procedures.

All infants were scanned in a 3 Tesla MRI magnet (Philips Ingenia) using a 32-channel head coil. High-resolution (1 mm) anatomical T2-weighted images and MPRAGE T1-weighted images were acquired, as well as susceptibility-weighted imaging. Infants with poor imaging quality were re-scanned; no participants had significant motion artifacts.

Brain abnormalities were defined using a standardized scoring system [30]. A single pediatric neuroradiologist, masked to clinical history, performed all qualitative and quantitative assessments of MR images. Abnormalities were graded on a scale between zero and four. Cerebral white matter abnormality was graded for: 1) cystic degeneration; 2) focal signal abnormalities; 3) delayed myelination; 4) thinning of the corpus callosum; 5) dilated lateral ventricles; and 6) reduction of white matter volume. Cortical gray matter abnormalities were graded for: 1) signal abnormality; 2) delayed gyration; 3) dilated extra-cerebral cerebrospinal fluid space. Deep nuclear gray matter abnormalities were graded for: 1) signal abnormality and 2) reduced deep gray matter volume. Cerebellar abnormalities were graded for: 1) signal abnormalities and 2) reduced cerebellar volume. All brain biometric scores were age corrected with a linear regression analysis [corrected brain measurement = measured brain measurement + slope (40–PMA)]. Additional details reported in Jain et al. [26].

#### Correlates of autism risk.

Caregivers completed the Adaptive Behavior Assessment System, Third Edition (ABAS-3) assessing adaptive behavior in Conceptual, Practical, and Social domains [31]. Higher ABAS-3 standard scores (*M* = 100, *SD* = 15) indicate better adaptive functioning.

Caregivers completed the 1½–5 (*n* = 279) or 6–18 year (*n* = 34) version of the Child Behavior Checklist (CBCL) [32], assessing maladaptive behavioral and emotional problems. Both generate Externalizing and Internalizing T-scores (*M* = 50, *SD* = 10); higher scores indicate maladaptive behaviors.

The preschool (*n* = 21) or second edition (*n* = 288) Behavior Rating Inventory of Executive Function (BRIEF) [33] was completed by caregivers to assess real-world EF behaviors. Both versions generate Inhibit, Shift, Emotional Control, Working Memory, Plan/Organize, and General Executive Composite (GEC) T-scores (*M* = 50, *SD* = 10); higher scores indicate worse EF. Children were also administered the Minnesota Executive Function Scale (MEFS) [34] assessing cognitive flexibility, working memory, and inhibitory control. The MEFS starts at an age-dependent level and adapts to each child’s abilities. An age-normed standard score (*M* = 100, *SD* = 15) is computed; higher scores indicate better EF.

Children were administered the Differential Abilities Scale, Second Edition (DAS-II) [35] yielding Verbal, Nonverbal, and General Conceptual Ability (GCA) standard scores (*M* = 100, *SD* = 15). Higher scores indicate better cognitive functioning.

### Procedures

Caregivers of infants born VPT were recruited in the NICU. If the infant met inclusion criteria, an MRI was scheduled at 41 ± 1 weeks PMA and information was collected. A demographically matched group of term-born 5-year-olds was recruited when the VPT group turned 5 years old. At 5-years (corrected age for VPT), caregivers completed the SCQ, ABAS-3, BRIEF, and CBCL and children were administered the DAS-II and MEFS.

### Statistical analysis

Descriptive statistics and correlations were computed to confirm assumptions for the subsequent analyses. To identify independent variables associated with VPT child with versus without an SCQ ≥ 15, we utilized Elastic Net (EN) logistic regression [36]. The EN procedure performs simultaneous variable selection and shrinkage; therefore, independent variables with non-zero regression coefficients were retained as a parsimonious set of candidate independent variables. In a subsequent step, the retained variables were re-estimated in an unpenalized logistic regression model to obtain interpretable effect estimates. For each independent variable, we calculated odds ratios (ORs), 95% confidence intervals (CI), and corresponding *p*-values (two-tailed) to quantify its association with the outcome [i.e., SCQ score consistent with an autism diagnosis or SCQ ≥ 15)]. See Supplementary Materials for linear regression exploring the continuous SCQ total score as the outcome. Independent variables included white matter, cortical gray matter, cerebellar, and deep nuclear gray matter abnormality scores, moderate-severe chorioamnionitis, pregestational/gestational diabetes, GA, birthweight (z-score), Apgar score <5 at 5 min, PMA, HDP, ROP, sepsis, breastmilk at NICU discharge, and BPD grade. Sex, social risk, and caregiver report of the number of autistic family members were also included due to their established association with risk for autism. To examine correlates of autism risk, chi square and independent samples t-tests were computed comparing VPT children with and without an elevated SCQ score ≥15 on the cognitive, adaptive, behavioral, and EF measures.

## RESULTS

More children born VPT (*n* = 28, 8.9%) had an SCQ score consistent with autism risk (i.e., SCQ ≥ 15) than term-born controls (*n* = 5, 2.9%; χ^2^ = 6.31, *p* = 0.012). The EN-regularized logistic regression model retained 15 of the 18 predefined independent variables as summarized in Table 2 (deep nuclear gray matter abnormalities, Apgar score <5 at 5 min, and breastmilk at NICU discharge were not retained). Using a significance threshold of *p* < 0.05, cerebellar abnormalities, moderate-severe chorioamnionitis, sex, and social risk emerged as most strongly associated with autism risk. Of the 50 infants who had moderate to severe cerebellar abnormalities (i.e., score >2; median = 2), 10 (20%) screened positive for autism risk based on SCQ ≥ 15. Among 263 infants with no or mild cerebellar abnormalities (score ≤2), 18 (6.8%) screened positive for autism risk. Among the 44 infants with positive moderate-severe histologic chorioamnionitis, 10 (22.7%) screened positive for autism risk. Among the 266 infants with no or mild histologic chorioamnionitis, 18 (6.8%) screened positive for autism risk. Of the 28 children at risk for autism (i.e., SCQ ≥ 15), 50% had moderate to severe cerebellar abnormalities (i.e., score >2) and 35.7% were coded as moderate-severe histologic chorioamnionitis. VPT children with an SCQ ≥ 15 had poorer adaptive functioning, worse EF, more behavior problems, and lower cognitive functioning than those with an SCQ < 15 (Table 3).

## DISCUSSION

Children born VPT were rated as having elevated autism traits and symptoms on the SCQ, with nearly 9% of the children having an SCQ score ≥15. In addition to male sex and higher social risk, cerebellar abnormalities and moderate-severe chorioamnionitis were independently associated with autism risk. We did not find evidence for other prenatal, perinatal, and neonatal risk factors to be significantly associated with an SCQ score ≥15.

Although we could not confirm an autism diagnosis, an SCQ ≥ 15 is predictive of autism diagnosis [6, 37] including in VPT children [9], and VPT children with an SCQ ≥ 15 evidenced significant functional impairments consistent with autism (i.e., adaptive behavior, EF, behavioral/emotional functioning, and cognitive functioning deficits). Adaptive deficits were most notable in the social and conceptual domains. The largest BRIEF effect sizes were observed for Shift, Emotional Control, and Working Memory. The Shift subscale has been identified as an early diagnostic marker for autism [38] and the Emotional Control elevation is consistent with the well-established correlation between autism and emotion dysregulation [38]. Autism has been associated with maladaptive internalizing and externalizing behaviors [39]. Caution interpreting these findings is warranted as difficulties in language, cognition, executive functioning and adaptive functioning may themselves contribute substantially to elevated SCQ scores.

The cerebro-cerebellar system is a critical hub for complex cognitive and socioemotional processes (e.g., emotional perception, theory of mind, and social prediction and timing such as anticipation of others’ actions or reactions) [40]. It actively shapes social intelligence, relies on neural networks connected to the cerebrum, and is especially vulnerable to inflammation disruptions early in development [40]. Thus, individuals with disruption to cerebellar development exhibit social disinhibition and impaired affect recognition and perspective taking, all of which are features associated with autism [41, 42]. Further, cerebellar abnormalities are associated with increased autism risk in VPT children. Specifically, cerebellar hemorrhages or lesions early after birth are associated with autism risk, particularly in severely premature, low birthweight infants [24]. Cerebellar hemorrhagic injury was significantly associated with an SCQ ≥ 15 in premature 3-year olds [8], and reduced cerebellar volumes were associated with critical-positive M-CHAT scores (i.e., high risk for autism) in toddlers [43] and with an autism diagnosis at age 7 [23]. Preterm birth and cerebellar hemorrhage may specifically disrupt a period of highly dynamic cerebellar development characterized by morphologic and synaptic reorganization that is more rapid in the third trimester, a critical time that is spent in the NICU by VPT infants [24]. Nonetheless, this finding may be due to other factors such as genetic liability, parental psychiatric history, maternal mental health, and postnatal environmental influences. Regardless of prematurity, the cerebellum is implicated in many of the behavioral symptoms in autism including repetitive behaviors, sensory perception, and cognitive ability [44].

Moderate-to-severe chorioamnionitis was associated with autism risk. This finding is consistent with studies reporting a high rate of chorioamnionitis in VPT infants associated with either abnormal scores on an autism screener or a definitive autism diagnosis [45]. Chorioamnionitis triggers a maternal and fetal inflammatory response, with pro-inflammatory cytokines crossing the placenta and entering fetal circulation [14]. This may negatively impact the fetal brain by causing abnormal synaptic pruning, impaired oligodendrocyte maturation, and/or disrupted myelination [14].

### Clinical and research implications

In addition to the known risk factors of family history of autism, male sex, and low socioeconomic status, the presence of these two novel risk factors (cerebellar abnormalities and moderate-severe histologic chorioamnionitis) should heighten a clinician’s suspicion for autism and result in earlier screening to enable early prevention/treatment of autism during periods of optimal neuroplasticity. Addressing disparities due to income, employment, and education may further help maximize neurodevelopmental outcomes. Further, these results underscore the multifactorial nature of autism risk in VPT children, involving both biological and social determinants, and highlight the importance of including multiple risk factors in exploring autism risk. The relation between maternal and fetal inflammation and autism risk should continue to be a focus of neurodevelopmental science. Additional work is needed to disentangle the association between moderate-severe chorioamnionitis and cerebellar abnormalities with autism risk.

### Limitations

Despite the large sample, longitudinal study design, and inclusion of multiple risk factors for autism, there are study limitations. Most notably, we could not determine whether children classified as at-risk for autism (i.e., SCQ ≥ 15) received a definitive autism diagnosis. Further, we did not have parallel information on early risk factors in the term-born children. Although we discuss the various risk factors as separable, they may co-occur and are likely related (e.g., social risk may interact with brain development). Also, the observed neonatal complications may index broader neurologic or systemic vulnerability associated with prematurity rather than represent specific mechanistic pathways to autism. It would be ideal to have neuroimaging data at the 5-year corrected age visit to understand how brain maturation and neuroplasticity contribute to autism risk.

### Conclusions

These findings expand previous research by examining longitudinal outcomes for VPT up to 5-years corrected age in the US, a longer follow-up period than most prior studies, but still early enough to facilitate timely intervention. Brain abnormalities, specific to the cerebellar region, and moderate-severe histologic chorioamnionitis were independently associated with autism risk in children born VPT, even when accounting for sex, social risk, and family history of autism. Early screening and intervention for preterm infants with these known risk factors, particularly targeting motor, executive functioning, and speech and language development, may help improve neurodevelopmental outcomes.

## Supplementary Material

Supplemental Materials

**Supplementary information** The online version contains supplementary material available at https://doi.org/10.1038/s41372-026-02856-x.

## Figures and Tables

**Table 1.** Demographic/clinical characteristics.

Characteristic	Very preterm (*n* = 315)	Term-born (*n* = 172)
Male	51.7%	50.6%
Race/Ethnicity		
White	*n* = 215 (68.3%)	*n* = 91 (52.9%)
Black	*n* = 83 (26.3%)	*n* = 61 (35.5%)
Hispanic	*n* = 22 (7.0%)	*n* = 10 (5.8%)
Asian	*n* = 8 (2.5%)	*n* = 3 (1.7%)
Other	*n* = 20 (6.3%)	*n* = 10 (5.9%)
Do not know/Refuse	*n* = 6 (1.9%)	*n* = 1 (0.6%)
Gestational age in weeks (M, SD)	29.2 (2.5)	39.3 (0.9)
Birthweight in grams (M, SD)	1276.4 (441.5)	n/a
Age at testing (M, SD)	69.5 (2.0) corrected	67.7 (1.9) chronological
Social risk score (M, SD)	2.9 (2.2)	2.6 (2.4)
Global brain abnormalities (M, SD)	6.15 (5.5)	n/a
Cerebellar abnormalities (M, SD)	1.28 (1.7)	n/a
Cortical gray matter abnormalities (M, SD)	1.01 (1.3)	n/a
White matter abnormalities (M, SD)	2.88 (2.9)	n/a
Deep nuclear gray matter abnormalities (M, SD)	0.95 (1.3)	n/a
Postmenstrual age at MRI (M, SD)	42.7 (1.3)	n/a
Moderate-severe histologic chorioamnionitis	14.0%	n/a
Apgar score<5 at 5 min	13.7%	n/a
Hypertensive disorders of pregnancy	41.9%	n/a
Retinopathy of prematurity	35.2%	n/a
Blood stream infection (sepsis)	11.4%	n/a
On breastmilk at NICU discharge	54.3%	n/a
Pregestational/gestational diabetes	17.1%	n/a
Bronchopulmonary dysplasia grade	0 = 55.2% 1 = 24.4% 2 = 16.2% 3 = 4.1%	n/a
Family history of autism	*n* = 82 (26.1%)	*n* = 42 (24.4%)

*n/a* not available for term-born controls.

Caregivers could select more than 1 race.

**Table 2.** Logistic regression assessing infant and maternal risk factors for autism in VPT children at 5-years corrected age.

SCQ ≥ 15			
χ^2^ = 61.16**			
Variable	OR	(95% CI)	*p*
Cerebellar abnormalities	1.339	(1.010, 1.778)	0.040*
Cortical gray matter abnormalities	1.111	(0.790, 1.540)	0.531
White matter abnormalities	1.013	(0.882, 1.162)	0.848
Moderate-severe histologic chorioamnionitis	4.148	(1.294, 13.368)	0.016*
Gestational age	0.870	(0.626, 1.199)	0.397
Birthweight (z-score)	0.829	(0.470, 1.422)	0.505
Postmenstrual age at MRI scan	0.872	(0.605, 1.258)	0.460
Hypertensive disorders of pregnancy	2.003	(0.688, 6.024)	0.205
Retinopathy of prematurity	1.088	(0.232, 4.966)	0.913
Sepsis	0.233	(0.039, 1.025)	0.075
Pregestational/gestational diabetes	2.435	(0.701, 7.982)	0.146
Bronchopulmonary dysplasia grade	1.163	(0.582, 2.295)	0.665
Sex	0.269	(0.082, 0.772)	0.02*
Social risk score	1.543	(1.241, 1.963)	0.000**
Number of autistic family members	1.537	(0.839, 2.685)	0.138

Sex was coded 1 = male, 0 = female.

*OR* Odds ratio, *CI* Confidence interval.

* *p* < 0.05,

** *p* < 0.01.

**Table 3.** Clinical correlates of autism risk (SCQ score ≥ 15) in VPT children at 5-year corrected age.

Correlate	SCQ < 15 Mean (*SD*)	SCQ ≥ 15 Mean (*SD*)	*t*-test	*d*
*Adaptive functioning*				
ABAS GAC	96.39 (11.44)	69.18 (16.10)	*t*(308) = 11.52, *p* < 0.001	2.0
ABAS conceptual	96.45 (11.01)	70.04 (14.96)	*t*(309) = 11.69, *p* < 0.001	2.0
ABAS social	99.66 (11.05)	75.86 (12.06)	*t*(309) = 10.79, *p* <0.001	2.1
ABAS practical	95.43 (12.75)	70.39 (17.82)	*t*(309) = 9.52, *p* < 0.001	1.6
*Executive functioning*				
BRIEF inhibit	56.15 (11.53)	64.11 (13.68)	*t*(308) = −3.42, *p* < 0.001	0.7
BRIEF shift	56.14 (12.03)	72.18 (12.85)	*t*(308) = −6.68, *p* < 0.001	1.3
BRIEF emotional control	56.52 (11.15)	69.18 (12.28)	*t*(308) = −5.68, *p* < 0.001	1.1
BRIEF working memory	55.72 (10.84)	67.64 (9.99)	*t*(308) = −5.59, *p* < 0.001	1.1
BRIEF plan/organize	50.21 (10.03)	59.36 (13.44)	*t*(308) = −4.45, *p* < 0.001	0.9
BRIEF GEC	54.36 (10.71)	70.67 (12.13)	*t*(308) = −8.36, *p* < 0.001	1.4
MEFS	94.99 (11.17)	75.13 (16.12)	*t*(300) = 7.89, *p* < 0.001	1.4
*Behavioral/emotional functioning*				
CBCL internalizing	50.80 (10.59)	63.74 (9.52)	*t*(309) = −6.12, *p* <0.001	1.3
CBCL externalizing	49.00 (11.38)	60.48 (13.81)	*t*(309) = −4.91, *p* <0.001	1.0
*Cognitive functioning*				
DAS GCA	94.60 (15.12)	58.50 (25.07)	*t*(311) = 11.23, *p* <0.001	1.7
DAS verbal	98.61 (16.64)	61.85 (29.35)	*t*(309) = 10.13, *p* <0.001	1.5
DAS nonverbal	97.37 (13.87)	67.43 (25.65)	*t*(311) = 9.91, *p* <0.001	1.5

*ABAS* Adaptive Behavior Assessment System, Third Edition, *GAC* General Adaptive Composite, *BRIEF* Behavior Rating Inventory of Executive Function Preschool or 2nd Edition, *GEC* General Executive Composite, *MEFS* Minnesota Executive Function Scale, *CBCL* Child Behavior Checklist, *DAS* Differential Abilities Scale, Second Edition, *GCA* General Conceptual Ability, *d* Cohen’s d *d* ≥ 8 is considered a large effect size.

## Data Availability

The study design, hypotheses, sample size, data collection, exclusion criteria and analysis plan for the primary study were preregistered on clinicaltrials.gov (NCT03345069). The analysis plan for the current study was not preregistered. The study data and statistical syntax that support the findings of this study can be requested from the corresponding or last author upon reasonable request and execution of a data sharing agreement.
