# Supplementary material for: Early autism risk factors: a cohort study of children born very preterm at 5-years
Source: J Perinatol. Author manuscript; Available in PMC 2026 Sep 1. (PMC13531389; doi:10.1038/s41372-026-02856-x)
Supplement: Supplementary file 1 [file NIHMS2203789-supplement-Supplemental_Materials.pdf]

Supplemental Materials

Stone-Heaberlin, M., Blackburn, A.D., Tamm, L., Allahverdy, A., Kline-Fath, B., & Parikh, N.A for the Cincinnati Infant Neurodevelopment Early Prediction Study (CINEPS) Investigators (2026). Early autism risk factors: A cohort study of children born very preterm at 5-years. Journal of Perinatology.

Contents

Summary of analytic approach with Social Communication Questionnaire (SCQ) total score – p. 2
Summary of results with SCQ score – p. 2
Supplemental Table 1: Linear regression results with SCQ total score – p. 3
References cited – p. 4

Summary of analytic approach for Social Communication Questionnaire (SCQ) total score:

To identify independent variables associated with an elevated SCQ score, we utilized Elastic Net (EN) linear regression [1]. The EN procedure performs simultaneous variable selection and shrinkage; therefore, independent variables with non-zero regression coefficients were retained as a parsimonious set of candidate independent variables. In a subsequent step, the retained variables were re-estimated in an unpenalized regression model to obtain interpretable effect estimates. Independent variables included white matter, cortical grey matter, cerebellar, and deep nuclear grey matter abnormality scores, moderate-severe chorioamnionitis, pregestational/gestational diabetes, GA, birthweight (z-score), Apgar score<5 at 5 minutes, PMA, HDP, ROP, sepsis, breastmilk at NICU discharge, and BPD grade. Sex, social risk, and caregiver report of the number of autistic family members were also included due to their established association with risk for autism.

Summary of results for SCQ total score:

The EN-regularized regression model retained 14 of the 18 predefined independent variables as summarized in Supplemental Table 1 (PMA, breastmilk at NICU discharge, birthweight, and retinopathy of prematurity were not retained). Using a significance threshold of $p<0.05$, family history of autism, sex, social risk, and hypertensive disorders of pregnancy emerged as most strongly associated with autism risk, while moderate-severe histologic chorioamnionitis was marginally significant. Notably, the SCQ total score was not normally distributed (M=6.88, SD=5.53; median = 5; skewness = 1.44, SE = .14; kurtosis = 2.54, SE = .28; significant Shapiro-Wilk test); therefore, results should be interpreted cautiously.

Supplemental Table 1

Linear Regression Assessing VPT Infant and Maternal Risk Factors for Autism Risk (Social Communication Questionnaire Total Score) at 5 Years Corrected Age

Predictor	β-Coefficient	(95% CI)	p
Moderate-severe histologic chorioamnionitis	1.54	(-0.06, 3.14)	$p=.059$
Gestational age	-0.15	(-0.45, 0.15)	$p=.335$
Apgar<5 at 5 minutes	0.68	(-0.93, 2.29)	$p=.409$
Hypertensive disorders of pregnancy	1.15	(0.04, 2.26)	$p=.042$
Sepsis	-1.56	(-3.44, 0.33)	$p=.105$
Pregestational/gestational diabetes	0.44	(-0.98, 1.86)	$p=.546$
Bronchopulmonary dysplasia grade	0.20	(-0.65, 1.06)	$p=.640$
Sex	-1.47	(-2.55, -0.38)	$p<.001$
Social risk score	0.81	(0.56, 1.05)	$p<.001$
Number of autistic family members	2.01	(1.17, 2.84)	$p<.001$
Cerebellar abnormalities	0.30	(-0.11, 0.71)	$p=.153$
Cortical grey matter abnormalities	0.35	(-0.09, 0.79)	$p=.121$
White matter abnormalities	0.20	(-0.04, 0.43)	$p=.102$
Deep nuclear grey matter abnormalities	0.28	(-0.31, 0.87)	$p=.345$

Note: Sex was coded 0=male, 1=female.

References cited

1. Zou H, Hastie T-. Regularization and variable selection via the elastic net. *Journal of the Royal Statistical Society Series B: Statistical Methodology* 2005, 67**:** 301-320.
